# Orthotropic Constitutive Modeling and Tsai–Wu Failure Criterion for Carbon Fiber-Reinforced PEEK Composites

**DOI:** 10.3390/polym17081076

**Published:** 2025-04-16

**Authors:** Yu Ye, Zixin Yang, Dianwei Qu, Bingyin Hu, Lei Li

**Affiliations:** 1 Key Laboratory of High Efficiency and Clean Mechanical Manufacture (Ministry of Education), School of Mechanical Engineering, Shandong University, Jinan 250061, China; 202214338@mail.sdu.edu.cn (Y.Y.); 202334466@mail.sdu.edu.cn (Z.Y.); 2 Shandong Wuzheng Group Co., Ltd., Rizhao 262300, China; dianwei.qu@wuzheng.com (D.Q.); bingyin.hu@wuzheng.com.cn (B.H.)

**Keywords:** carbon fiber-reinforcement, PEEK, orthotropic constitutive modeling, Tsai–Wu failure criterion

## Abstract

This paper performs orthotropic constitutive modeling for short carbon fiber-reinforced polyetheretherketone (CF-PEEK) composites fabricated using material extrusion 3D printing technology. A variety of specimens for tensile, compressive, and shear tests are 3D printed under different deposition path patterns. The related experimental results disclose the strong directional mechanical properties, including tensile/compressive modulus and strength. The Tsai–Wu failure criterion is also developed based on the experimental data. The tensile–compressive behavior models of different orthotropic materials were constructed by importing the experimental data into COMSOL, followed by a compression simulation of the S-shaped specimen. The experimental results of the S-shaped compression test were compared with the COMSOL-based simulation analysis, which validated the effectiveness of the Tsai–Wu failure prediction. The predicted failure timings, locations and load–displacement curves all show a good agreement with experimental observations. Furthermore, the Tsai–Wu failure index is incorporated as a stress constraint in structural topology optimization, showing the effect of significantly reduced stress concentration. These findings and data will be supportive for the design and optimization of 3D printed CF-PEEK composites.

## 1. Introduction

Additive manufacturing (AM), also known as 3D printing, has rapidly evolved in recent years [1]. Among its various techniques, material extrusion-based 3D printing, which extrudes molten material filaments from a heated nozzle, has become the most popular method for thermoplastics, due to its material versatility, cost-effectiveness, and ease of implementation [2]. One notable development is the growing interest in high-performance materials like polyetheretherketone (PEEK) and its composites [3,4]. The exceptional thermal stability, strength, bio-compatibility, and chemical resistance make PEEK ideal for various applications in aerospace, automotive, and medical industries [5,6,7]. The adoption of fiber reinforcement enhances the aforementioned excellent properties. Current research on models for PEEK and carbon fiber-reinforced PEEK (CF/PEEK) primarily focuses on thermal behavior and elastoplastic behavior modeling. Liu et al. [8] developed a thermal field model for high-speed milling of CF/PEEK, accounting for thermal anisotropy and nonlinear conductivity. Yan et al. [9] proposed a temperature model for grinding CF/PEEK composites, incorporating material anisotropy. Liu et al. [10] introduced an anisotropic elastoplastic model for SCFR-PEEK under biaxial loading. Lei et al. [11] created a 3D thermomechanical model for stretch-induced anisotropy in PEEK.

However, despite its many benefits, material extrusion of CF-PEEK composites faces challenges that hinder the realization of the high-level mechanical performances of the printed parts. One of the primary issues is anisotropy [12,13], which refers to the variation in material properties depending on the load orientation. This anisotropic behavior is problematic in structural applications where design with isotropic material models is generally assumed, meaning that performing anisotropy-aware design is particularly necessary [14,15,16,17].

The orientation of fibers within PEEK composites is a key factor contributing to the anisotropy of AM parts [18]. The positioning of fibers, whether they are aligned in a specific direction or randomly distributed, directly influences the part’s strength and stiffness [19]. Controlling fiber orientation during the printing process is critical for tailoring the mechanical properties of the final product [20,21,22]. Proper fiber alignment can significantly enhance the load-bearing capacity and overall structural integrity, while improper orientation can lead to weak points and poor performance in certain loading conditions [23].

In addition to fiber orientation, another critical factor that influences the anisotropic mechanical performance of AM parts is the interlayer bonding [24,25]. The materials are deposited layer by layer, and the layers are generally weakly bonded [24,26]. This weak interlayer adhesion may lead to failure under shear stress, limiting the durability and strength of the part. Strengthening the interlayer bonding is therefore a crucial aspect when enhancing the overall mechanical performance of AM parts [27,28].

Given the above illustration, obtaining an understanding of the anisotropic material model of CF-PEEK composites and deeply incorporating it in structural analysis and design is necessary when seeking to ensure consistency between design and manufacture. Experiments to construct the orthotropic constitutive model on material extrusion-based thermoplastics have been widely conducted [29,30,31,32], most particularly for ABS and PLA materials, from which the factors of print parameters, print pattern, and meso-structures have all been determined to influence the orthotropic properties. With regard to strength modeling, although the Tsai–Wu criterion has the limitation of not effectively distinguishing between different failure modes, it is still widely used to predict failure in composite materials. This is due to its simplicity in calculation, particularly with regard to fiber-reinforced laminates [33,34,35] and 3D printed samples [36,37]. The Tsai–Wu criterion accounts for the interactions between different stress components, making it effective for analyzing strengths under multi-axial stresses [38], which is particularly useful for anisotropic materials, as it allows for the transformation of stress components to align with the symmetry axes under complex loading conditions. Research on the integration of stress-constrained topology optimization with additive manufacturing has attracted increasing attention [12,39]. However, most studies in the literature employ the conventional von Mises stress as the constraint indicator, which fails to capture the failure behavior of anisotropic structures [40], meaning that the Tsai–Wu failure criterion is more suitable in this regard.

Topology optimization (TO) aims to optimize structures by eliminating excess materials and preserving those essential for load paths, and includes the solid isotropic materials with penalization(SIMP) method [41], the bi-directional evolutionary structural optimization (BESO) method [42], the level set method [43], and the smooth-edged material distribution for optimizing topology (SEMDOT) method [44]. With the advancement of AM technology, the TO design of composite materials, which have traditionally posed manufacturing challenges, has become feasible [45,46]. The integration of TO with AM has already led to numerous successful cases [47,48].

As summarized above, most of existing research involving the orthotropic constitutive modeling and Tsai–Wu failure criterion has focused on composite laminates with carbon fibers or 3D printed ABS/PLA/nylon materials, with limited investigations on CF-PEEK composites manufactured using the material-extrusion additive manufacturing process. Given the strengths of CF-PEEKs in a variety of industries, this study intends to present a comprehensive study on the orthotropic modeling of mechanical properties for CF-PEEKs, disclosing the process-related reasons causing the material anisotropy. It then seeks to incorporate the orthotropic constitutive model and the Tsai–Wu failure model in simulation-based analysis, the result of which will be verified by experiments. The collected data, the material property models, and the procedures of analysis would all inspire the community to push forward the applications of CF-PEEKS. The remainder of the paper is organized as follows. Section 2 introduces the experimental preparation and establishes a coordinate system to describe specimens printed with different deposition paths. Section 3 discusses the experimental results of the tensile, compressive, and shear tests. This is followed by a comparison of COMSOL Multiphysics software (V 6.2) simulations and experimental validation in Section 4. Section 5 validates the effectiveness of the Tsai–Wu failure index in reducing stress concentrations through topology optimization. The manuscript ends with concluding remarks in Section 6.

## 2. Theoretical Principles and Experimental Preparation

### 2.1. Characteristics of the Material Extrusion AM Process

**Figure 1:**
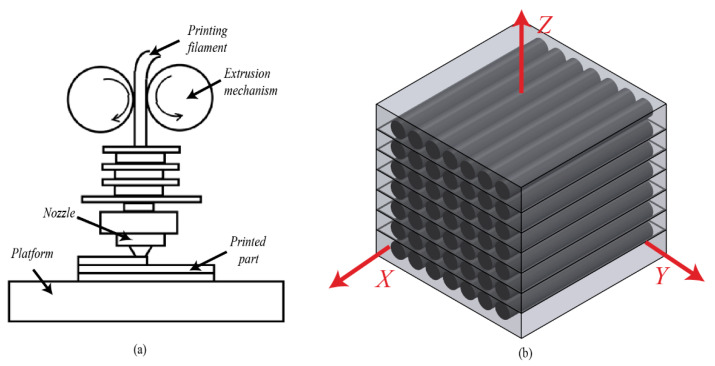
Illustration of the material extraction process: (**a**) schematic diagram of the material extrusion mechanism, (**b**) coordinate system for CF-PEEK modeling.

In the material extrusion AM process, the orthotropic properties of the printed samples primarily result from the arrangement of printing paths and the interlayer bonding characteristics. As shown in Figure 1a, the AM system builds parts layer by layer through fused filament deposition, where materials are extruded through a nozzle to form continuous filaments. The bonding effect between extruded filaments is influenced by the melting and cooling behavior of the thermoplastic materials. The directional solidification, as well as the filament deposition-induced meso-scale pores, both result in orthotopically distinctive mechanical properties. To facilitate further analysis, a local coordinate system is defined in this study, as shown in Figure 1b, and as follows:

- The X-direction represents the filament deposition direction, corresponding to the printing path.
- The Y-direction is perpendicular to the filament deposition direction that reflects the intralayer bonding between fibers.
- The Z-direction represents the build direction that indicates the interlayer bonding.

In the following sections, a series of experiments are designed and conducted to characterize the orthotropic material model of CF-PEEK composites.

### 2.2. Sample Preparation

In this study, a 10 wt% short carbon fiber (CF)-reinforced PEEK composite material, with 25 L/D (length to diameter) ratio and which was provided by Yimai Company (Dongguan, China), was used. The samples were printed using a MAGIC-HT-M (Yimai, Dongguan, China) 3D printer.

The printing parameters are as follows: layer thickness is 0.2 mm, nozzle temperature is 450 °C, printing speed is 10 mm/s, and fill density is 100%. To ensure high interlayer bonding quality, the printing platform temperature was maintained at 180 °C. Other printing parameters are provided in Table 1, below.

### 2.3. Experiment Design

**Figure 2:**
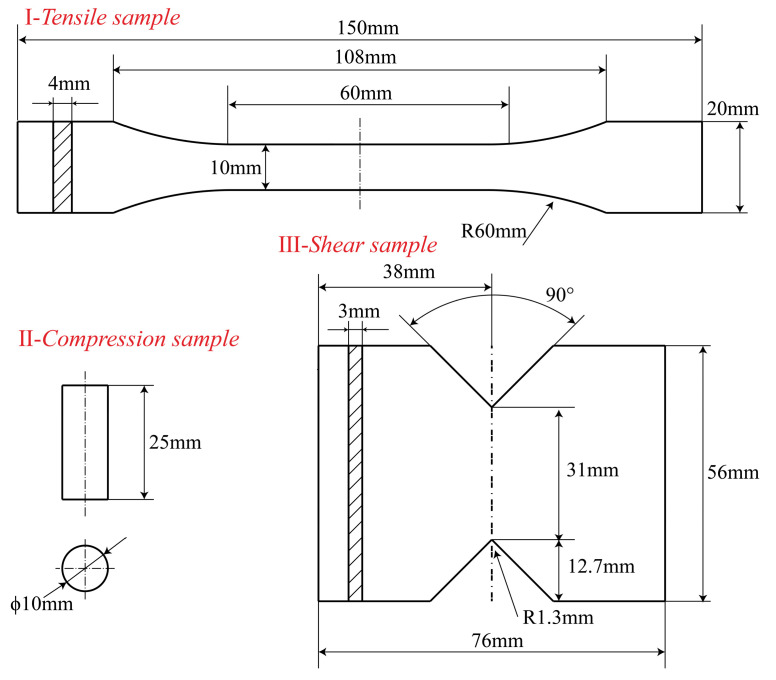
Test samples: I—tensile specimen, II—compression specimen, III—shear specimen.

In accordance with the ISO 527-4 [49], ASTM D7078 [50], and GB/T 1448 [51] standards, the CF-PEEK composite test samples were fabricated. The detailed dimensions of the samples are shown in Figure 2.

The tensile, shear, and compression tests were conducted at room temperature (25 °C). A computer-controlled universal testing machine (WDW-10, Zhongluchang, Jinan, China) equipped with a 100 kN force sensor was used to measure the tensile strength, compressive strength, elastic modulus, and Poisson’s ratio at a loading rate of 2 mm/min. Strain gauges were employed to measure the horizontal and vertical strains of the specimens, from which the elastic modulus and Poisson’s ratio were calculated. Specialized fixtures were prepared for shear strength and shear modulus testing, following the ASTM D7078 standard. Finally, the microstructures at the tensile fracture surfaces were observed using a scanning electron microscope (SEM, JSM-7610F, JEOL, Beijing, China) to analyze internal defects and fracture mechanisms.

**Figure 3:**
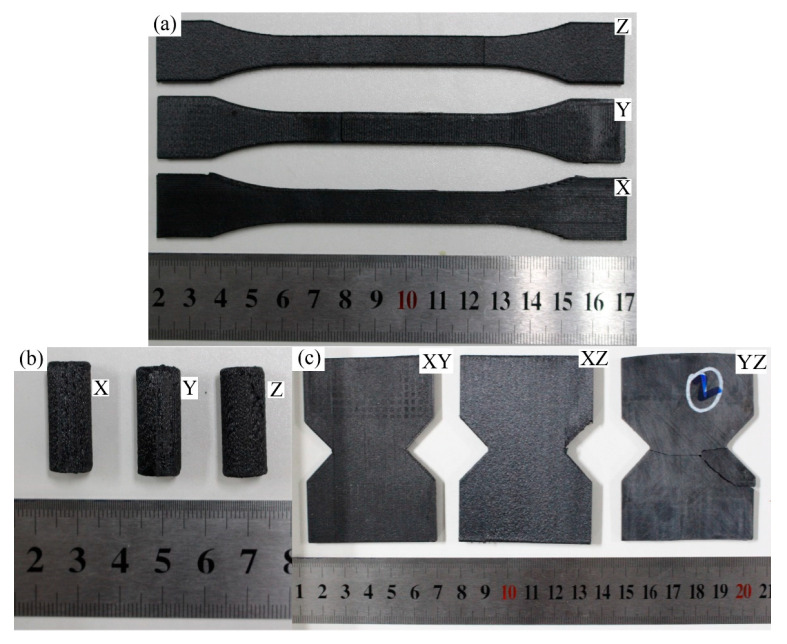
The mechanical testing specimens and dimensions made by CF-PEEK composites. (**a**) tensile specimen; (**b**) compression specimen; (**c**) shear specimen.

**Figure 4:**
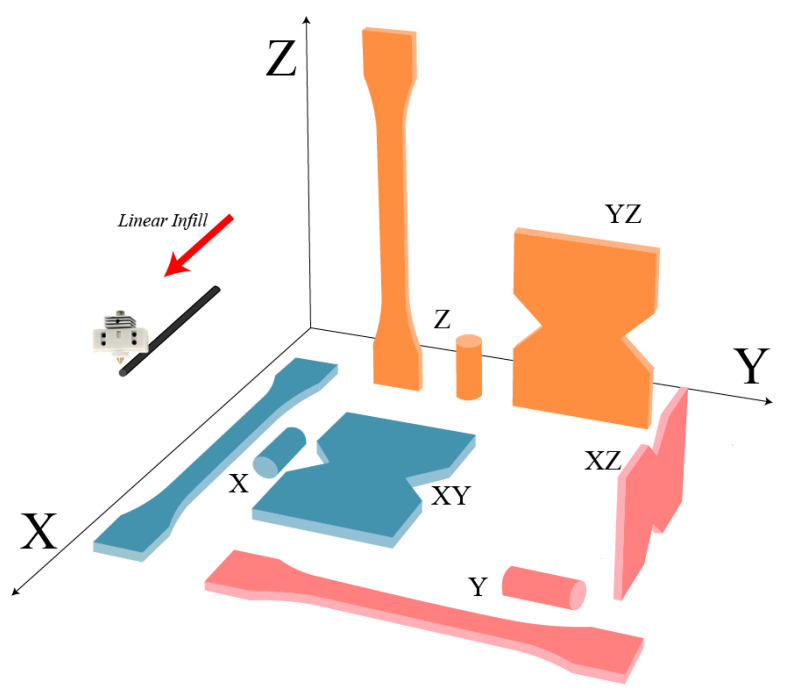
The printing arrangement of the specimens with blue-filament direction aligned with the loading direction, red-filament direction aligned with the perpendicular direction to the loading, and orange- filament direction aligned with the building direction.

In order to investigate the directional properties, three types of printing arrangements are assigned to each sample, as shown in Figure 3 and Figure 4. Accordingly, each test, tensile, compressive, or shear, includes three types of specimens, labeled as X, Y, and Z, respectively.

For instance, the tensile specimen, shown with blue color in Figure 4, has the filament direction aligned with the loading direction, indicated by the X-type tensile specimen, and the tensile specimen in red has its filament direction aligned with the perpendicular direction to the loading, named by the Y-type tensile specimen. The experiments were repeated two~three times for each type of specimen in order to check the repeatability of results. The experimental data are presented as averages with deviations.

## 3. Experimental Results and Discussion

### 3.1. Tensile Test Result Analysis

**Figure 5:**
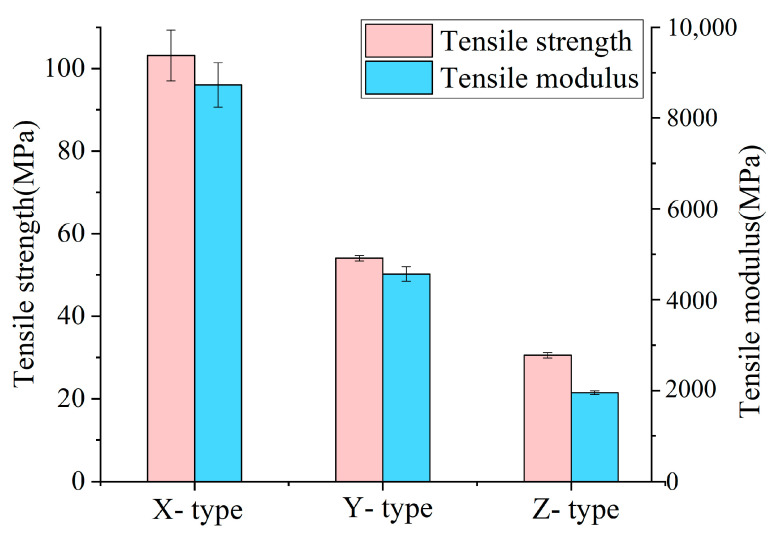
The tensile strength and tensile modulus of the printed CF-PEEK tensile specimens.

**Figure 6:**
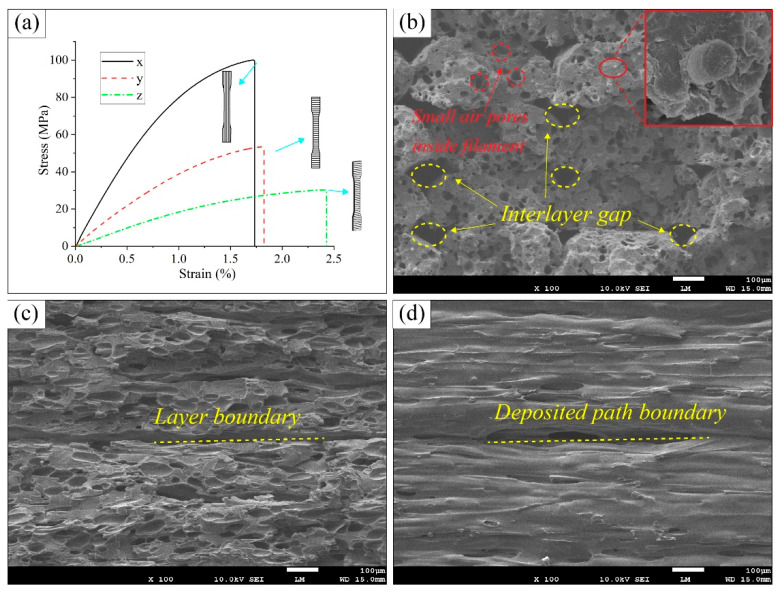
Tensile stress–strain curves and fracture topography of the printed CF-PEEK tensile specimens. (**a**) Stress–strain curves of the printed CF-PEEK tensile specimens; (**b**) SEM fracture surface of the X-type specimen; (**c**) SEM fracture surface of the Y-type specimen; (**d**) SEM fracture surface of the Z-type specimen.

Figure 5 presents the tensile mechanical properties of the printed CF-PEEK composites. Among the various groups of tensile specimens, the X-type specimens exhibited the most pronounced fiber reinforcement effect, with a tensile strength of 103.15 MPa and a tensile modulus of 8730.11 MPa. As shown in Figure 6b, the interlayer bonding of the X-type specimen is moderate, with the presence of porosities and air void defects. All of these pores at the fusion boundary are potential origins of cracks [5,38], which may cause fast crack propagation in the tensile loading process [52]. However, because of the alignment of the fiber direction and the tensile stress direction, the contribution of the fiber-reinforcement phase is maximized. The fracture surface reveals a brittle fracture mode.

The tensile strength of the Y-type specimens decreased significantly, to only 54.4 MPa, with a tensile modulus of 4566.67 MPa. As shown in Figure 6c, the fracture does not occur along the interfilament interface, indicating good interfilament bonding. However, because the fiber reinforcement direction is perpendicular to the tensile stress direction, the matrix materials bear the majority of the load with rare fiber reinforcement effect.

The Z-type specimens exhibited the weakest tensile performance, with a tensile strength of only 30.53 MPa and a tensile modulus of 1952.67 MPa. As seen in Figure 6d, the interlayer bonding is poor, and the insufficient interlayer bonding strength resulted in the delamination failure of the specimen.

In conclusion, both the printing path and fiber orientation significantly influence the mechanical properties of the CF-PEEK composites. Optimizing fiber orientation distribution while enhancing the inter- and intra-layer filament bonding will be crucial to improving the mechanical properties.

### 3.2. Compression Test Result Analysis

**Figure 7:**
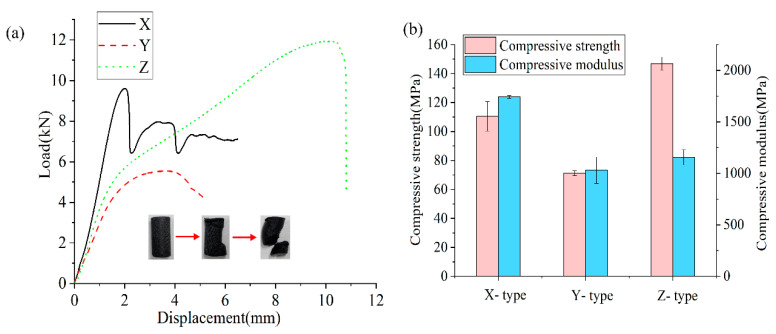
Compressive load–displacement curves and the related compressive strength and modulus data. (**a**) Load–displacement curve of the CF-PEEK compression specimens. (**b**) Compressive strength and compressive modulus of the CF-PEEK compression specimens.

The Z-type specimens, printed vertically (i.e., along the build direction), exhibited the highest compressive strength, with an average of approximately 146.86 MPa (Figure 7b) and a compressive modulus of 1155.70 MPa. The high strength can primarily be attributed to the compressive load being applied perpendicular to the interlayer bonding, which significantly reduces the direct impact of the interlayer bonding’s fragility. Under the compressive loading, shear failure typically occurs at a 45-degree angle, and this typical failure mode indicates that, even with weak interlayer bonding, the overall structure can effectively withstand compressive forces in the vertical direction. Furthermore, the load–displacement curve exhibits evident plastic deformation, showing that, after the initial linear elastic phase, the material can continue to bear more compressive loads instead of fracturing immediately. This plastic deformation behavior suggests that the interlayer bonding is sufficient to resist compressions through high energy absorption and dissipation.

The compressive strength and modulus of the X-type specimens (see Figure 7b) were 110.46 MPa and 1742.38 MPa, respectively. Due to the high strength and stiffness of carbon fibers, even under the shear failure mode at a 45-degree angle, the fibers and the matrix can withstand a rather large compressive load, exhibiting a large compressive modulus. However, the toughness is poor, as shown by the load–displacement curve, where the material’s performance rapidly deteriorates after reaching the peak.

The compressive strength and modulus of the Y-type specimens show the lowest value of 71.23 MPa and 1031.15 MPa, respectively. In this group, the fiber orientation was perpendicular to the loading direction, and the load bearing capacity primarily depended on the interfilament bonding. However, the meso-scale pores at the interfilament interface degraded the deformation-resistance of the specimen, resulting in a low compressive modulus. Under compression, delamination or tearing between layers is prone to occur, and the insufficient interlayer bonding strength leads to limited plastic deformation in the Y-type compressive specimens.

### 3.3. Shear Test Result Analysis

**Figure 8:**
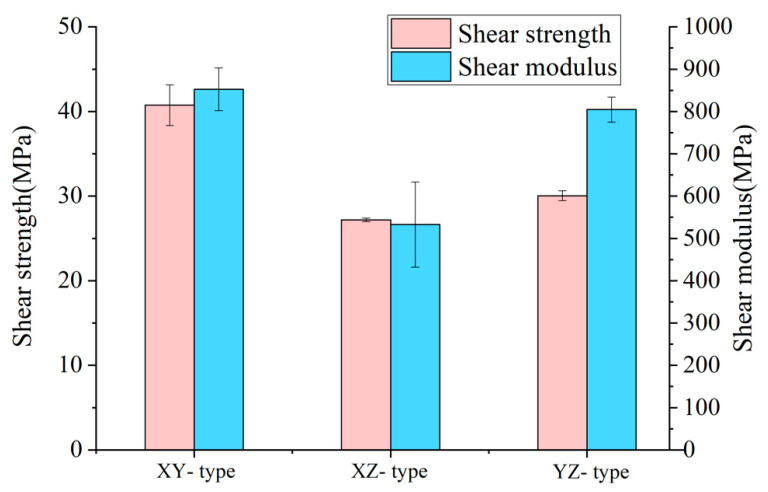
The shear strength and shear modulus of the printed CF-PEEK shear specimens.

As shown in Figure 8, the shear strength of the XY specimens is 40.74 MPa, with a shear modulus of 852.35 MPa; the shear strength of the XZ specimens is 27.19 MPa, with a shear modulus of 532.58 MPa; and the shear strength of the YZ specimens is 30.05 MPa, with a shear modulus of 804.64 MPa. These results reveal significant differences in directional shear performances.

**Figure 9:**
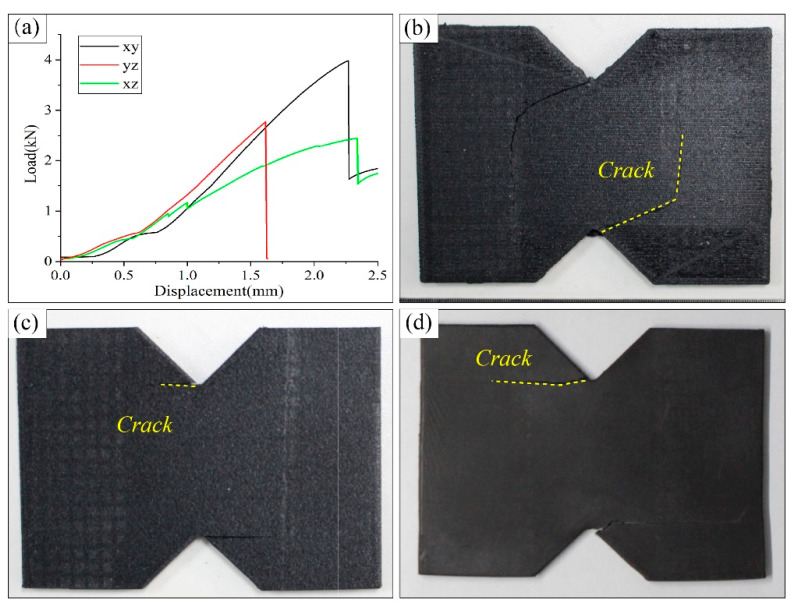
Tensile load–displacement curves and fracture images of the printed CF-PEEK shear specimens. (**a**) Load–displacement curve of the printed CF-PEEK shear specimens; (**b**) failure crack of the XY-type specimen; (**c**) failure crack of the XZ-type specimen; (**d**) failure crack of the YZ-type specimen.

The differences in shear failure behaviors of the three types of specimens can be clearly identified from the load–displacement curve in Figure 9a The load–displacement curves for the XY and YZ specimens exhibit a similar failure process: upon reaching the peak shear stress, the curve experiences a sharp drop, indicating that these specimens undergo direct crack failure after shear damaging. In contrast, the curve for the XZ specimens rises gradually, demonstrating that the XZ specimens exhibit a nonlinear stiffness degradation, with cracks progressively opening as the fixture moves, indicating a slow process of crack propagation.

Additionally, the crack morphology in Figure 9b–d provides some further insights about the failures. The XY specimens fail directly at the stress concentration spot with a slashed crack path, demonstrating strong shear resistance owing to the firm bonding of filaments inside a printing layer. The XZ specimens, however, show rapid crack propagation initiated from the notch tip and extending rapidly along the interlayer interface, attributed to insufficient interlayer bonding strength. In contrast, the YZ specimens exhibit a cross-section composed of purely filament bonding, along the build direction or along the in-layer transverse direction. The strengths of both directions are relatively lower than the in-layer filament direction, and, hence, the cracks are prone to expand, though there is a long process of gradual crack extension before total failure is encountered.

### 3.4. Elastic Modulus and Poisson’s Ratio

It is challenging to describe the plastic behavior of printed materials using only elastic modulus constants, since both the compressive and tensile moduli differ significantly with strains. Therefore, when performing part-scale analysis while taking into account the tensile–compressive anisotropy and plasticity, the experimental tensile and compressive stress–strain curves for X- and Y-type samples are directly used. This is achieved by fitting the stress–strain relationship and incorporating it into the COMSOL analysis. For an exception, the elastic modulus constants were adopted for direction Z, as non-obvious variations were identified.

In this study, the tensile and compressive experimental data were fitted separately. During subsequent simulations, the stress state of each grid cell is determined based on the sign of the strain. A positive strain indicates the cell under tension and the tensile modulus function that is applied, while a negative strain indicates the cell under compression, and the compressive modulus function is used. It is also important to note that both the tensile and compressive moduli were smoothly treated with C0 continuity at zero strain.

**Figure 10:**
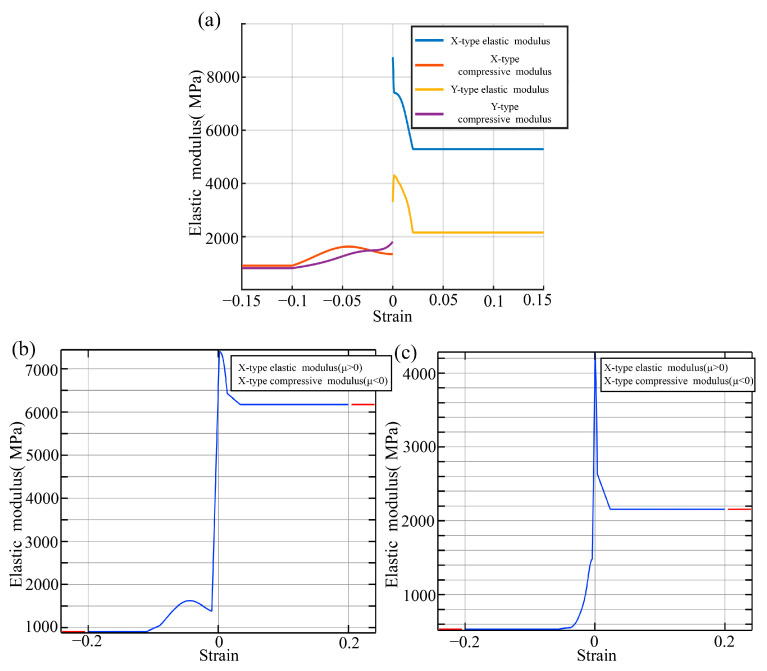
Elastic modulus–strain relationship curves. (**a**) Original modulus curves against the tensile and compressive strains along the X and Y axes; (**b**) X-direction tensile and compressive modulus curves after continuity treatment; (**c**) Y-direction tensile and compressive modulus curves after continuity treatment.

The following four formulas fit the X-direction tensile modulus, X-direction compressive modulus, Y-direction tensile modulus, and Y-direction compressive modulus using Gaussian functions (normal distribution functions). The fitting function coefficients are listed in Table 2. Each fitted parameter (e.g., $X_{a1}$, $X_{b1}$, $X_{c1}$) is accompanied by a 95% confidence interval. The fitted curves are plotted in Figure 10.

(1) $$E_{XT}=\sum_{i=0}^{5}X_{ai}exp\left(-{\left(\frac{\varepsilon_{x}-X_{bi}}{X_{ci}}\right)}^{2}\right)$$

(2) $$E_{XC}=\sum_{i=0}^{6}x_{ai}exp\left(-{\left(\frac{\varepsilon_{x}-x_{bi}}{x_{ci}}\right)}^{2}\right)$$

(3) $$E_{YT}=\sum_{i=0}^{4}Y_{ai}exp\left(-{\left(\frac{\varepsilon_{y}-Y_{bi}}{Y_{ci}}\right)}^{2}\right)$$

(4) $$E_{YC}=\sum_{i=0}^{5}y_{ai}exp\left(-{\left(\frac{\varepsilon_{y}-y_{bi}}{y_{ci}}\right)}^{2}\right)$$

## 4. Establishment of the Simulation Model and Experimental Comparison

### 4.1. Failure Evaluation Model for CF-PEEK

Due to the anisotropic nature of composite materials, their elastic modulus and strength vary significantly in different directions. For CF-reinforced composites, research has proposed various failure criteria to describe their damage and failure standards. Among the most commonly applied criteria are the maximum stress criterion, the maximum strain criterion, and the Tsai–Wu criterion [53].

The Tsai–Wu failure criterion is an important failure criterion in the structural analysis of composite materials. It distinguishes the stress state of the composite materials and describes the material’s overall failure behavior through a polynomial function of the stress components. The specific formula for Tsai–Wu failure criterion is given in Equation (5). This criterion introduces material constants, including $F_{1},\;\;F_{2},F_{11},F_{22},F_{33},F_{44},F_{55},F_{66}$, and interaction terms, including $F_{12},F_{23},F_{13}$, fully reflecting the individual and interactive effects of multiple stress components (including normal stress and shear stress) to describe the anisotropic and tensile–compressive distinctive failure potentials.

The Tsai–Wu criterion formula is as follows:

(5) $$F_{1}\sigma_{x}+F_{2}\sigma_{y}+F_{3}\sigma_{z}+F_{11}\sigma_{x}^{2}+F_{22}\sigma_{y}^{2}+F_{33}\sigma_{z}^{2}+F_{44}\tau_{yz}^{2}+F_{55}\tau_{xz}^{2}+\;F_{66}\tau_{xy}^{2}+2F_{12}\sigma_{x}\sigma_{y}+2F_{23}\sigma_{y}\sigma_{z}+2F_{13}\sigma_{x}\sigma_{z}\leq 1$$

where $\sigma_{x},\sigma_{y},\sigma_{z}$ are the three normal stresses of the material; $\tau_{xy},\tau_{yz},\tau_{xz}$ are the three shear stresses.

(6) $$F_{1}=\frac{1}{X_{T}}-\frac{1}{X_{C}},F_{2}=\frac{1}{Y_{T}}-\frac{1}{Y_{C}},F_{3}=\frac{1}{Z_{T}}-\frac{1}{Z_{C}}$$

(7) $$F_{11}=\frac{1}{X_{C}X_{T}},F_{22}=\frac{1}{Y_{C}Y_{T}},\;F_{33}=\frac{1}{Z_{C}Z_{T}},$$

(8) $$F_{44}=\frac{1}{S_{yz}^{2}},F_{55}=\frac{1}{S_{xz}^{2}},F_{66}=\frac{1}{S_{xy}^{2}}\;\;$$

(9) $$F_{12}=-\frac{1}{2}\sqrt{F_{11}F_{22}},F_{23}=-\frac{1}{2}\sqrt{F_{22}F_{33}},F_{13}=-\frac{1}{2}\sqrt{F_{11}F_{33}}$$

$X_{T}$ and $X_{C}$, $Y_{T}$ and $Y_{C}$, $Z_{T}$ and $Z_{C}$ are the uniaxial tensile and compressive yield strength in the X, Y, and Z directions, respectively; $S_{xy}$, $S_{yz}$, $S_{xz}$ are the shear strengths in the XY, YZ, and XZ planes, respectively.

When the calculated value from Equation (5) is greater than or equal to 1, it is determined that the material fails. The Tsai–Wu criterion comprehensively considers the combined effects of normal stresses and shear stresses on failure, making it particularly suitable for strength analysis of composite materials under complex stress states.

Based on the experimental data obtained in the previous section, the final strength parameters are summarized in Table 3.

### 4.2. Simulation Model Establishment

Unlike the commonly used L-beams with clearly defined failure locations, the selection of the S-shaped structure provides generality with an unknown failure location. The S-shaped structure was chosen to validate the accuracy of failure criteria by comparing experimental and simulation results to determine if the failure locations align.

**Figure 11:**
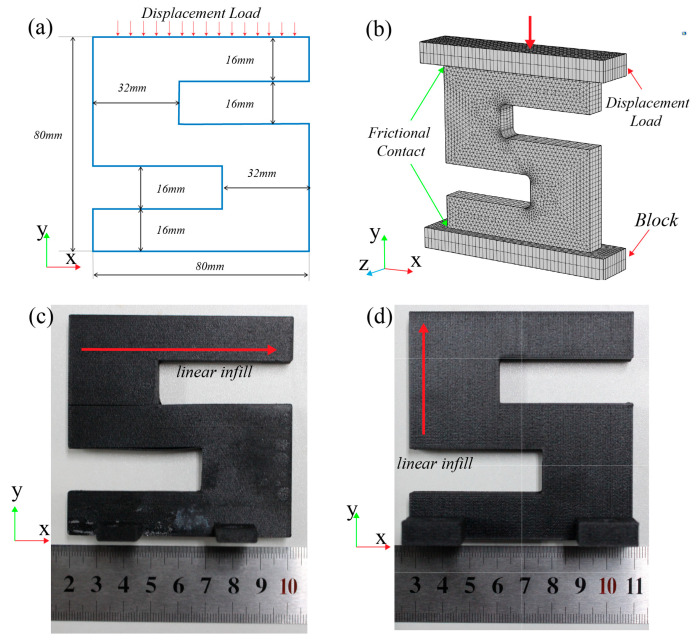
The part for simulation-based verification of the Tsai–Wu failure model. (**a**) The S-shaped part with its dimensions; (**b**) finite element model; (**c**) X-type sample part; (**d**) Y-type sample part.

The S-shaped structural model for simulation is shown in Figure 11a. The total height of the part is 80 mm, the total width is 80 mm, and the thickness is 10 mm. Other specific dimensions are also provided in the figure. The top of the part is in contact with a compression block, and a displacement load of 5 mm is applied along the Y-direction using this compression block. The loading step is set to 0.1 mm to simulate the compression deformation process. The bottom of the specimen is in contact with another support block, and the bottom surface of this block is fully fixed, constraining all degrees of freedom to prevent displacement or rotation during the loading process.

The upper and lower surfaces of the S-shaped part are allowed to slide horizontally, with friction considered. A face-to-face contact model is employed to simulate friction between the contact surfaces. Coulomb’s friction model is used to calculate the frictional force, with a friction coefficient of 0.3. As shown in Figure 11c,d, in order to assess the suitability of the Tsai–Wu failure criterion under different printing conditions and complex loading scenarios, the S-shaped part is categorized into X-type and Y-type based on the printing path direction. Both types of sample parts are placed directly on the universal testing machine for compressive testing.

To accurately capture the stress concentration effects and improve computational precision, the COMSOL Multiphysics^®^ Software (V 6.2) was used to generate an initial tetrahedral mesh for the entire specimen, with the overall mesh density set to extremely fine. We generated a mesh with 12,610 elements for use in future studies. The maximum element size in the chosen mesh is 2 mm, and the minimum element size is 0.05 mm. Then, local mesh refinement was applied to the corner regions of the S-shaped specimen. These regions, due to abrupt geometric changes, tend to exhibit significant stress concentrations. The refined mesh enhances the numerical solution accuracy in these critical areas. Furthermore, a sweeping meshing strategy was employed for the contact pairs, thereby increasing the contact analysis accuracy, and also improving the numerical convergence stability of the nonlinear contact analysis. The final meshing result and boundary conditions are shown in Figure 11b. The simulation applied solid mechanics and domain differential equations, accounting for geometric nonlinearity and utilizing a steady-state solver.

### 4.3. Comparison of Experimental and Simulation Results

Based on the anisotropic and tensile–compressive asymmetric constitutive models established in the previous sections, as well as the experimentally determined Tsai–Wu failure criterion, this section presents a detailed finite element analysis (FEA) and evaluation of the compressive performance of the X-type and Y-type sample parts using COMSOL Multiphysics^®^. During simulation, when the Tsai–Wu failure index of a mesh element reaches 1, that element is removed from the result visualization. This approach provides an intuitive representation of the progressive failure process.

**Figure 12:**
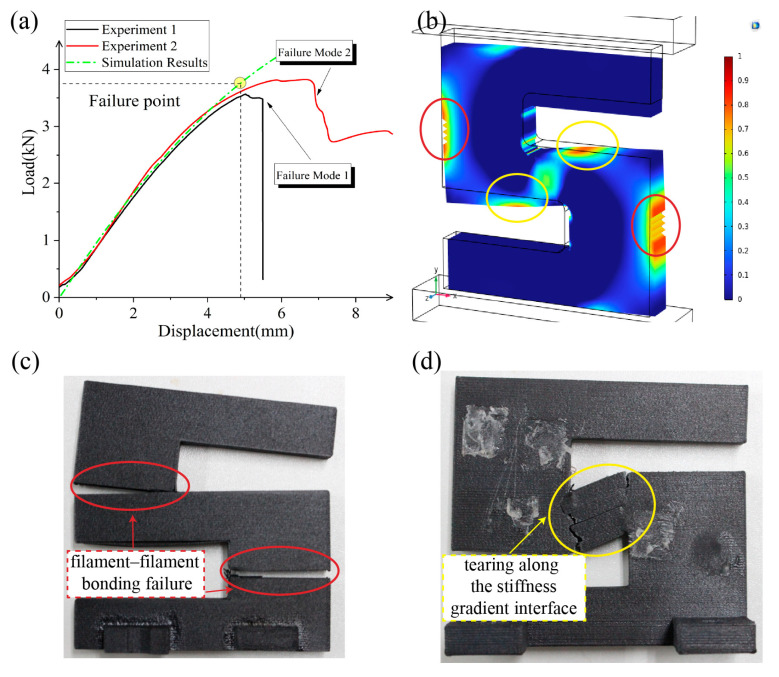
Comparison between test data and simulation results. (**a**) Compressive load–displacement curve of the X-type sample parts; (**b**) Tsai–Wu failure index map of the X-type sample part; (**c**) failure mode 1; (**d**) failure mode 2.

Figure 12 presents the compression simulation and experimental results for the X-type sample parts.

The simulation predicts failure at a compression displacement of 4.9 mm, with a corresponding reaction force of 3.75 kN. When compared with the experimental curve, the simulation-predicted structural stiffness and maximum load capacity show an acceptable agreement, with the simulated nonlinear behavior slightly different than the actual experimental results. In experiment 2 in particular, the curve keeps elongating after the predicted failure point, reflecting the insufficient modeling of plasticity. Notably, and as shown in Figure 12c,d, the same type X-type sample parts exhibit two distinctly different failure modes. Failure mode 1, depicted in Figure 12c, shows that the Y-direction tensile stress at the marked location (red circle) exceeds the yield strength, resulting in an instantaneous tensile fracture of the experimental part. This represents filament–filament bonding failure. Failure mode 2, shown in Figure 12d within the yellow circle, occurs when the combined X-direction tensile stress and XY-plane shear stress exceed the yield strength, causing a sudden stiffness change and prolonged tearing along the stiffness–gradient interface.

**Figure 13:**
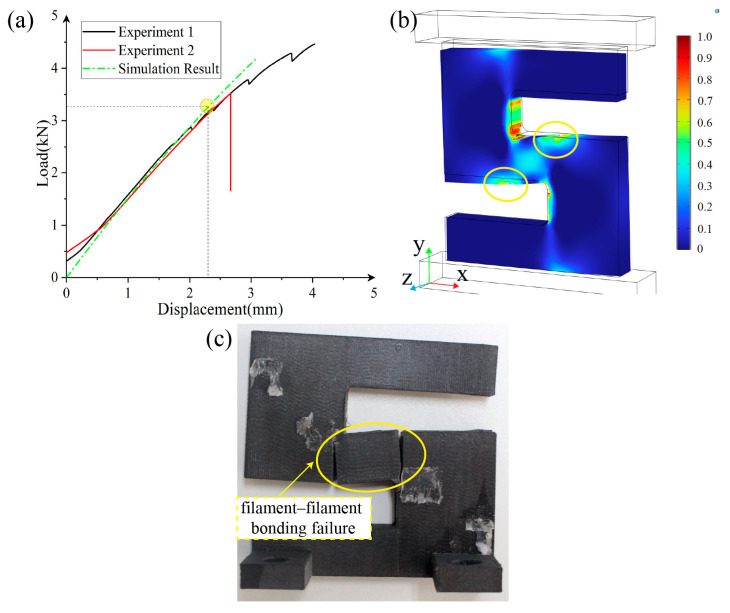
Comparison between test data and simulation results. (**a**) Compressive load–displacement curve of the Y-type sample parts; (**b**) Tsai–Wu failure index map of the Y-type sample part; (**c**) failure mode demonstration.

As shown in Figure 13a, the simulation result for the Y-type sample part indicates that failure occurs at a compression displacement of 2.3 mm, with a reaction force of 3.32 kN. The Y-type experimental part exhibits only one failure mode, as indicated by the yellow circle in Figure 13c, where the combined X-direction tensile stress and XY-plane shear stress exceed the yield strength, leading to local filament–filament debonding and an instantaneous tearing after the peak load. Comparing the load–displacement curve, the simulated model predicts a higher structural stiffness and maximum load capacity than the experimental values. The structural stiffness is close to the experimental measurement, and the compressive strength is slightly lower than that measured in Experiment 2, though it demonstrates a more significant gap when compared with the measurement in Experiment 1. These performance differences between the simulation and experimental results, as well as between the experimental results themselves, reflect the inconsistencies in processing quality in material extraction-based 3D printing.

To summarize, comparing the experimental and simulation results reveals good consistency in the failure timing, location, and load–displacement curve, indicating that the experimental obtainment of the Tsai–Wu failure criterion for 3D printed CF-PEEK parts through material extrusion is valid.

## 5. Topology Optimization Based on Orthogonal Anisotropy with Tsai–Wu Failure Constraint

So far, we have established the orthotropic elasticity model and the Tsai–Wu failure criterion for CF-PEEK, while a direct application is to support strength-constrained topology optimization, enhancing the structural performance of 3D printed CF-PEEK components. The paper uses the SIMP method, and extensive applications have demonstrated its reliability and maturity. The specific algorithm is briefly introduced below.

### 5.1. Material Interpolation Method

The design variable for each element, denoted as $\rho_{e}$, is constrained within the interval [0, 1], where 0 corresponds to a void and 1 represents a full solid element. To prevent intermediate values, a penalization function is applied. The SIMP method is employed for stiffness interpolation, where the stiffness of an element is defined as follows:

(10) $$E_{e}=E_{min}+{\bar{\rho }}_{e}^{p}\left(E_{0}-E_{min}\right)$$

Here, p represents the penalization factor (typically set to 3), $E_{0}$ is the stiffness of the solid material, and $E_{min}$ is a very small value (chosen as 10^−12^) used to prevent singularities of the stiffness matrix.

To avoid artificially low stresses in low density areas, a distinct exponent q (e.g., q = 0.5) is introduced for stress interpolation. Specifically, the stress is interpolated as follows:

(11) $$\sigma_{e}=\left(\varepsilon +{\bar{\rho }}_{e}^{q}\left(1-\varepsilon \right)\right)D_{0}B_{e}u_{e}$$

where ε is a very small value (set to 10^−9^), $D_{0}$ represents the material elasticity matrix that defines the constitutive relationship, $B_{e}$ is the element’s strain–displacement matrix, and $u_{e}$ is the corresponding nodal displacement vector.

Consequently, the element stress vector, consisting of six components, is assembled as follows:

(12) $$\sigma_{e}={\left[\sigma_{ex},\sigma_{ey},\sigma_{ez},\tau_{eyz},\tau_{exz},\tau_{exy}\right]}^{T}$$

Subsequently, the Tsai–Wu failure criterion is applied to the stress vector of the element through a weighted summation, resulting in a scalar stress index, denoted as $\sigma_{TW,e}$:

(13) $$\sigma_{TW,e}=F_{1}\sigma_{ex}+F_{2}\sigma_{ey}+F_{3}\sigma_{ez}+F_{11}\sigma_{ex}^{2}+F_{22}\sigma_{ey}^{2}+F_{33}\sigma_{ez}^{2}+F_{44}\tau_{eyz}^{2}\;\;\;+F_{55}\tau_{exz}^{2}+F_{66}\tau_{exy}^{2}+2F_{12}\sigma_{ex}\sigma_{ey}+2F_{23}\sigma_{ey}\sigma_{ez}+2F_{13}\sigma_{ex}\sigma_{ez}$$

The constitutive matrix $D_{0}$ for a three-dimensional orthotropic material is expressed as follows:

(14) $$D_{0}=\left[\begin{array}{cccccc} \frac{1-\mu_{yz}\mu_{zy}}{E_{y}E_{z}\Delta } & \frac{\mu_{yx}+\mu_{zx}\mu_{yz}}{E_{y}E_{z}\Delta } & \frac{\mu_{zx}+\mu_{yx}\mu_{zy}}{E_{y}E_{z}\Delta } & 0 & 0 & 0\\ \frac{\mu_{xy}+\mu_{xz}\mu_{zy}}{E_{x}E_{z}\Delta } & \frac{1-\mu_{zx}\mu_{xz}}{E_{x}E_{z}\Delta } & \frac{\mu_{zy}+\mu_{zx}\mu_{xy}}{E_{x}E_{z}\Delta } & 0 & 0 & 0\\ \frac{\mu_{xz}+\mu_{xy}\mu_{yz}}{E_{x}E_{y}\Delta } & \frac{\mu_{yz}+\mu_{xz}\mu_{yx}}{E_{x}E_{y}\Delta } & \frac{1-\mu_{xy}\mu_{yx}}{E_{x}E_{y}\Delta } & 0 & 0 & 0\\ 0 & 0 & 0 & G_{yz} & 0 & 0\\ 0 & 0 & 0 & 0 & G_{xz} & 0\\ 0 & 0 & 0 & 0 & 0 & G_{xy} \end{array}\right]$$

(15) $$\Delta =\frac{1-\mu_{xy}\mu_{yx}-\mu_{zx}\mu_{xz}-\mu_{yz}\mu_{zy}-2\mu_{xy}\mu_{yz}\mu_{zx}}{{E_{x}E}_{y}E_{z}}$$

(16) $$D_{0}=\left[\begin{array}{cccccc} 10131 & 2195.9 & 1690.4 & 0 & 0 & 0\\ 1899.6 & 5188.0 & 1128.9 & 0 & 0 & 0\\ 1125.7 & 1093.1 & 2310.6 & 0 & 0 & 0\\ 0 & 0 & 0 & 800 & 0 & 0\\ 0 & 0 & 0 & 0 & 500 & 0\\ 0 & 0 & 0 & 0 & 0 & 850 \end{array}\right]$$

In this case, $E_{x}$, $E_{y}$, and $E_{z}$ are assigned values of 9.0 GPa, 4.5 GPa, and 2.0 GPa, respectively, while the remaining parameters are specified in the aforementioned Table 3.

In stress-constrained optimization, directly using the “maximum local stress” as a constraint, or equivalently, ensuring that the maximum $\sigma_{PN}$ across all elements does not exceed 1, leads to objective and constraint functions that are discontinuous and non-differentiable, thereby presenting difficulties in numerical solution methods. A preferred approach is to use clustering functions to build a single global function that effectively quantifies the maximum stress value, for example, the p-norm function:

(17) $$\sigma_{PN}={\left(\sum_{e=1}^{N}{\left(\sigma_{TW,e}\right)}^{P}\right)}^{\frac{1}{P}}\leq 1$$

where $\sigma_{PN}$ is the global P-norm measure, P is the aggregation parameter, and N is the total number of elements.

### 5.2. Optimization Problem and Sensitivity Analysis

The design problem with Tsai–Wu failure constraint and volume constraint is formulated as follows:

(18) $$\left\{\begin{array}{c} \begin{array}{c} \min:C=U^{T}\mathrm{KU}=\sum_{e=1}^{N}u_{e}^{T}k_{e}u_{e}=\sum_{e=1}^{N}E_{e}u_{e}^{T}k_{0}u_{e}\\ s.t.:\left\{\begin{array}{c} KU=F\\ \begin{array}{c} \sigma_{PN}\leq 1\\ \begin{array}{c} \frac{\sum_{e=1}^{N}{\bar{\rho }}_{e}}{N}\leq V_{f} \end{array} \end{array} \end{array}\right. \end{array}\\ where:\rho_{min}\leq \forall {\bar{\rho }}_{e}\leq 1 \end{array}\right.$$

The objective function represents the strain energy of the structure, i.e., the compliance C. The first constraint corresponds to the structural equilibrium (stiffness) equation, the second constraint represents the Tsai–Wu failure constraint, and the third constraint is the volume constraint. F is the global load vector, U is the global displacement vector, and K is the global stiffness matrix. Additionally, $u_{e}$ is the displacement vector for an individual element, $k_{e}$ is its stiffness matrix, and $k_{0}$ is the stiffness matrix corresponding to a full solid element. $V_{f}$ denotes the volume fraction limit. The sensitivity of the objective function is well known and thus omitted here [54]. The sensitivity analysis of the below P-norm stress constraint is highlighted. The P-norm aggregates the many local constraints into a global one.

The measurement of stress is undertaken by the P-norm formulated using the Tsai–Wu stress. After obtaining the gradient of this model, the method of moving asymptotes (MMA) is adopted to update the topological configuration iteratively. Hence, details of sensitivity analysis are presented in the following. The sensitivity of the P-norm stress is given by the following:

(19) $$\frac{\partial \sigma_{PN}}{\partial \rho_{j}}=\sum_{e=1}^{N}\frac{\partial \sigma_{PN}}{\partial {\bar{\sigma }}_{TW,e}}\left[{\left(\frac{\partial {\bar{\sigma }}_{TW,e}}{\partial {\bar{\sigma }}_{e}}\right)}^{T}\frac{\partial \eta \left(\rho_{e}\right)\sigma_{e}}{\partial \rho_{j}}\right]\;=\sum_{e=1}^{N}\left(\frac{\partial \sigma_{PN}}{\partial {\bar{\sigma }}_{TW,e}}\left[{\left(\frac{\partial {\bar{\sigma }}_{TW,e}}{\partial {\bar{\sigma }}_{e}}\right)}^{T}\frac{\partial \eta \left(\rho_{e}\right)}{\partial \rho_{j}}\sigma_{e}\right]+\frac{\partial \sigma_{PN}}{\partial {\bar{\sigma }}_{TW,e}}\left[{\left(\frac{\partial {\bar{\sigma }}_{TW,e}}{\partial {\bar{\sigma }}_{e}}\right)}^{T}\eta \left(\rho_{e}\right)\frac{\partial \sigma_{e}}{\partial \rho_{j}}\right]\right)$$

The equations above can be rewritten in the following form:

(20) $$\left\{\begin{array}{c} \frac{\partial \sigma_{PN}}{\partial \rho_{j}}=A+B\\ \begin{array}{c} T_{1}=\sum_{e=1}^{N}\left(\frac{\partial \sigma_{PN}}{\partial {\bar{\sigma }}_{TW,e}}\left[{\left(\frac{\partial {\bar{\sigma }}_{TW,e}}{\partial {\bar{\sigma }}_{e}}\right)}^{T}\frac{\partial \eta \left(\rho_{e}\right)}{\partial \rho_{j}}\sigma_{e}\right]\right)\\ T_{2}=\sum_{e=1}^{N}\left(\frac{\partial \sigma_{PN}}{\partial {\bar{\sigma }}_{TW,e}}\left[{\left(\frac{\partial {\bar{\sigma }}_{TW,e}}{\partial {\bar{\sigma }}_{e}}\right)}^{T}\eta \left(\rho_{e}\right)\frac{\partial \sigma_{e}}{\partial \rho_{j}}\right]\right) \end{array} \end{array}\right.$$

Here, the following holds:

(21) $$\frac{\partial \sigma_{PN}}{\partial \sigma_{TW,e}}={\left(\sum_{e=1}^{N}{\left(\sigma_{TW,e}\right)}^{P}\right)}^{\frac{1}{P}-1}\cdot {\left(\sigma_{TW,e}\right)}^{P-1}$$

Based on the Tsai–Wu stress defined in Equation (13), the derivative of the local element Tsai–Wu failure criterion $\partial {\bar{\sigma }}_{TW,e}$ with respect to the stress vector ${\bar{\sigma }}_{e}$ can be written in the following form:

(22) $${\left(\frac{\partial {\bar{\sigma }}_{TW,e}}{\partial {\bar{\sigma }}_{e}}\right)}^{T}=\left[\begin{array}{c} F_{1}+2F_{11}{\bar{\sigma }}_{ex}+2F_{12}{\bar{\sigma }}_{ey}+2F_{13}{\bar{\sigma }}_{ez}\\ F_{2}+2F_{22}{\bar{\sigma }}_{ey}+2F_{12}{\bar{\sigma }}_{ex}+2F_{23}{\bar{\sigma }}_{ez}\\ \begin{array}{c} F_{3}+2F_{33}{\bar{\sigma }}_{ez}+2F_{23}{\bar{\sigma }}_{ey}+2F_{13}{\bar{\sigma }}_{ex}\\ 2F_{44}{\bar{\tau }}_{eyz}\\ \begin{array}{c} 2F_{55}{\bar{\tau }}_{exz}\\ 2F_{66}{\bar{\tau }}_{exy} \end{array} \end{array} \end{array}\right]$$

The analytical form of term $\partial \sigma_{e}/\partial \rho_{j}$ can be expressed as follows:

(23) $$\frac{\partial \sigma_{e}}{\partial \rho_{j}}=D_{0}B_{e}\frac{\partial u_{e}}{\partial \rho_{j}}=D_{0}B_{e}L_{e}\frac{\partial U}{\partial \rho_{j}}$$

Substituting Equation (23) into term $T_{2}$ in Equation (20), we find that $T_{2}$ can be rewritten as follows:

(24) $$T_{2}=\sum_{e=1}^{N}\left(\eta \left(\rho_{e}\right)\frac{\partial \sigma_{PN}}{\partial {\bar{\sigma }}_{TW,e}}{\left(\frac{\partial {\bar{\sigma }}_{TW,e}}{\partial {\bar{\sigma }}_{e}}\right)}^{T}D_{0}B_{e}L_{e}\frac{\partial U}{\partial \rho_{j}}\right)$$

The adjoint method is used to solve the $\partial U/\partial \rho_{j}$ in Equation (24). Specifically, taking derivatives of both sides of KU=F yields the following:

(25) $$\frac{\partial K}{\partial \rho_{j}}U+K\frac{\partial U}{\partial \rho_{j}}=0$$

Subsequently, the following expression is derived as below:

(26) $$T_{2}=\sum_{e=1}^{N}\left(-\eta \left(\rho_{e}\right)\frac{\partial \sigma_{PN}}{\partial {\bar{\sigma }}_{TW,e}}{\left(\frac{\partial {\bar{\sigma }}_{TW,e}}{\partial {\bar{\sigma }}_{e}}\right)}^{T}D_{0}B_{e}L_{e}\right)K^{-1}\frac{\partial K}{\partial \rho_{j}}U$$

Here, λ represents the adjoint variable, which is obtained by solving the following adjoint equation:

(27) $$K\lambda =\frac{\partial \sigma_{PN}}{\partial {\bar{\sigma }}_{TW,e}}{\left(\frac{\partial {\bar{\sigma }}_{TW,e}}{\partial {\bar{\sigma }}_{e}}\right)}^{T}\left(\frac{\partial \eta \left(\rho_{e}\right)}{\partial {\bar{\rho }}_{j}}\cdot D_{0}B_{e}L_{e}\right)$$

In Equation (27), $L_{e}$ is the global-to-local transformation matrix, and $u_{e}=L_{e}U$ represents the corresponding transformation.

**Figure 14:**
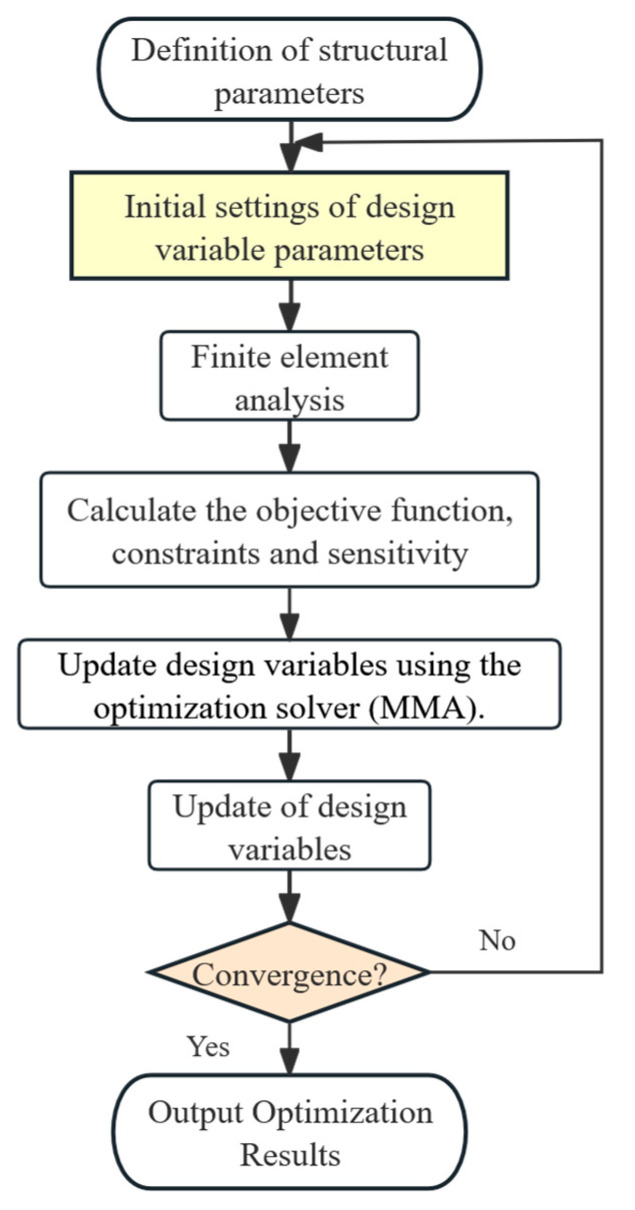
Diagram of the optimized numerical implementation process.

To avoid checkerboard patterns and enhance optimization stability, the typical smoothing filter and Heaviside projection are employed, for which the details are omitted for being well known [55]. The optimization steps for this study are shown in Figure 14.

### 5.3. L-Shaped Bracket Structure

**Figure 15:**
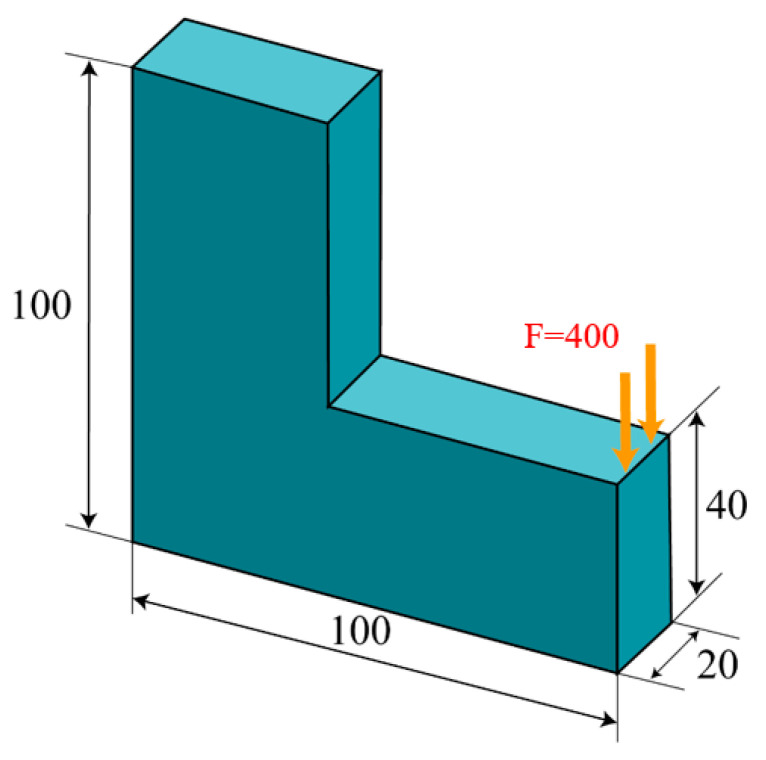
Dimensions and boundary conditions of the three-dimensional L-shaped bracket structure (units: mm).

This sub-section describes the performance of the optimization on a three-dimensional L-bracket structure. The boundary conditions and design domain are illustrated in Figure 15. The top surface of the L-bracket is fixed, while a uniformly distributed load (F = 400 N), is applied along the upper edge of the lower rectangular block. The design domain is discretized using 1 × 1 × 1 hexahedral element, with a volume fraction constraint of 0.3. The p-norm parameter is set to P = 20, and a filter radius of 3.5 times the element size is used to avoid checkerboard patterns. Convergence is achieved when the change in the objective function is less than 0.1% over five consecutive iterations, with the optimization continuing for at least 150 iterations.

**Figure 16:**
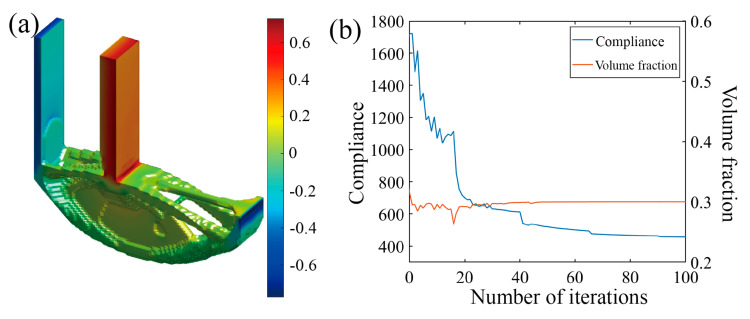
Optimization results of L-scaffold considering Tsai–Wu failure index as constraint. (**a**) Optimization result model; (**b**) optimized iterative curve.

**Figure 17:**
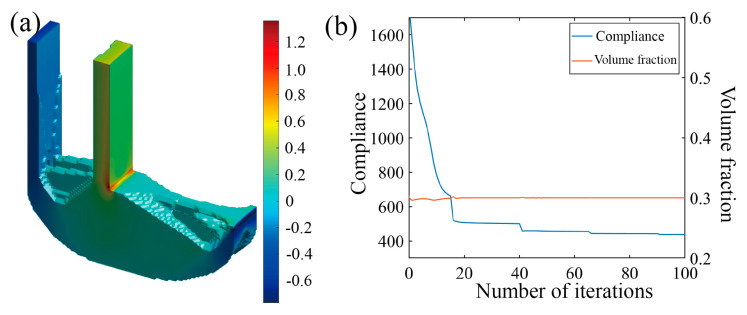
Optimization results of L-scaffold without considering Tsai–Wu failure index as constraint. (**a**) Optimization result model; (**b**) optimized iterative curve.

Two distinct optimization scenarios are examined, with or without incorporating the Tsai–Wu failure constraint, leading to different Tsai–Wu index distribution patterns. From the iteration curves in Figure 16b and Figure 17b, it can be observed that the optimization process is relatively smooth, and that the volume constraints in both microstructures are well satisfied. As depicted in Figure 16 and Figure 17, the optimization results present two different Tsai–Wu index contours, both of which have the stress concentrations localized at the re-entrant corner. By incorporating the Tsai–Wu constraint, the maximum index is controlled below 1, being approximately 0.67, owing to the rounding effect of the re-entrant. However, the maximum Tsai–Wu index reaches 1.36 if not specially managed.

### 5.4. The MBB Beam with a Notch

**Figure 18:**
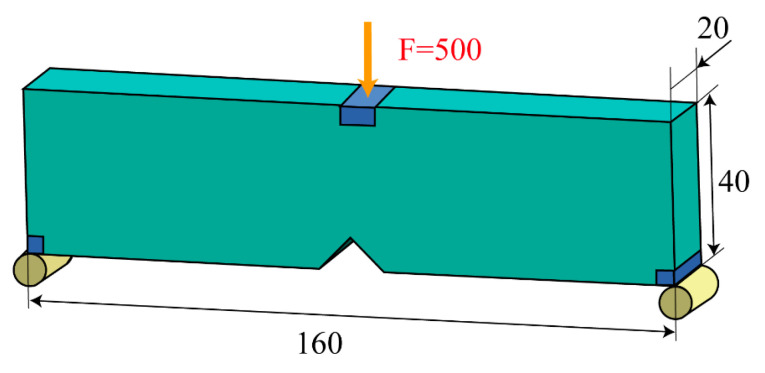
Dimensions and boundary conditions of the MBB beam with a notch (units: mm).

This section applies the proposed method to the notched MBB beam problem. As depicted in Figure 18, the initial design domain contains a notch that causes stress concentration. The two corners at the left and right ends are fixed (with zero vertical displacement), and a characteristic load of F = 500 N is applied. Similar to the previous case, the design domain is discretized using a 1 × 1 × 1 hexahedral element, with a volume fraction constraint of 0.2. The p-norm parameter is set to P = 50, and a filter radius of 3.5 times the element size is employed, while the convergence criteria and the number of optimization iterations remain the same as the last case.

**Figure 19:**
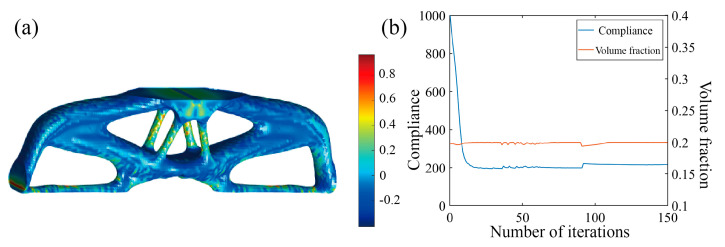
Optimization results of MBB beam with notch considering Tsai–Wu failure index constraints. (**a**) Optimization result model; (**b**) optimized iterative curve.

**Figure 20:**
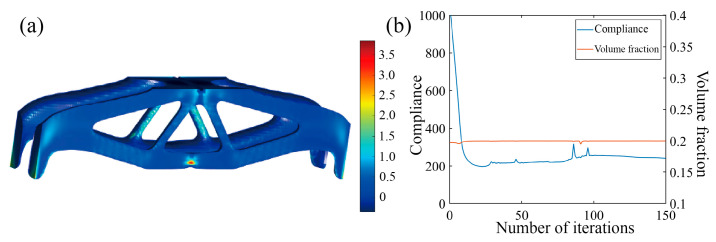
Optimization results of MBB beam with notch without Tsai–Wu failure index constraint. (**a**) Optimization result model; (**b**) optimized iterative curve.

Again, two optimization scenarios are analyzed, with or without incorporating the Tsai–Wu failure constraint, and the results are shown in Figure 19 and Figure 20. In the first scenario, the Tsai–Wu failure index contour tends to be uniform and features a maximum Tsai–Wu index of 1. In the second scenario, the Tsai–Wu failure index reaches 2.85 at the notch, indicating a highly concentrated stress region. These examples demonstrate that incorporating the Tsai–Wu failure index into optimization effectively improves stress distribution uniformness and reduces stress concentration in critical, failure-prone areas.

## 6. Conclusions

This paper primarily focuses on studying the mechanical properties and failure behavior of short carbon fiber-reinforced polyetheretherketone (CF-PEEK) composites under various loading conditions. The Tsai–Wu failure criterion was developed and validated through a combined approach of experimental testing and simulation. Specimens were fabricated using material extrusion 3D printing technology, and the tensile, compressive, and shear tests were designed and conducted, following relevant experimental standards.

The tensile test results indicate that fiber orientation has a significant influence on mechanical properties. The tensile test results indicate that fiber orientation significantly affects the mechanical properties, with the highest tensile strength and modulus observed along the longitudinal filament direction. In the X-type samples, where fibers are predominantly aligned with the printing direction, tensile strength reached 103.15 MPa and the modulus was as high as 8730.11 MPa. In contrast, the Y-type samples, characterized by fibers oriented perpendicular to the load direction, exhibited markedly lower tensile performance, recording a tensile strength of only 54.4 MPa and a modulus of 4566.67 MPa. Similarly, the Z-type samples showed the weakest tensile properties, with a tensile strength of 30.53 MPa and a modulus of 1952.67 MPa, due to the inefficient alignment of fibers for load bearing in tension. Compression tests revealed that the specimens printed in the build direction—corresponding to the Z-type—exhibited the highest compressive strength of 146.86 MPa, primarily owing to the compressive load application mitigating the adverse effects of insufficient interlayer bonding. Moreover, the Y-type samples, which rely mainly on the matrix and intralayer bonding, demonstrated lower performance in both compressive and shear tests, as interfacial defects led to early crack initiation. Based on the experimental results, the following recommendations are made with regard to selecting printing path models: The X-type model is recommended for applications requiring high overall mechanical performance, as it offers excellent tensile and compressive strength, making it an ideal choice for load-bearing structures in practice. The Z-type model excels in compressive applications. Despite weaker tensile and shear properties, it still provides high compressive strength, although potential issues with weak interlayer bonding should be noted. In shear tests, significant differences in shear performance were observed across different types of specimens, with the highest strength in the XY-type specimen (in-layer shear load) and the weakest for the YZ specimen, attributed to variations in interfacial bonding quality.

The Tsai–Wu failure criterion model was verified through simulation, and simulation analysis of the S-shaped sample part with different printing path directions was performed. The simulation successfully replicated the failure modes and mechanical behaviors observed in the experiments. The simulation results show good consistency with experimental data, particularly in terms of failure locations and load–displacement curves.

This study demonstrates that the material extrusion process significantly influences the mechanical properties of CF-PEEKs, and the Tsai–Wu failure criterion is effective in describing the performance of CF-PEEK composites under complex stress states.

The sensitivity-based topology optimization method, built on the established orthotropic constitutive model and Tsai–Wu failure criterion, applies the Tsai–Wu failure index to build the stress constraint. The optimized designs significantly reduce stress concentrations and enhance load capacity, validating the effectiveness of the improved composite structure performance. These findings provide both theoretical and experimental support for the design and optimization of 3D printed composites.

## Figures and Tables

**Table 1:** Material extrusion printing parameters for CF-PEEK composites.

Printing Parameters	Value
Nozzle diameter (mm)	0.4
Nozzle temperature (℃)	450
Platform temperature (℃)	180
Layer thickness (mm)	0.2
Printing speed (mm/s)	10
Infill density (%)	100
Wall thickness (mm)	0.4
Infill pattern	Lines
Overlap interval (mm)	0

**Table 2:** Gaussian fitting function coefficients.

Parameter	Values	Parameter	Values	Parameter	Values
$X_{a1}$	1.34 × 10^3^	$X_{b1}$	9.25 × 10^−6^	$X_{c1}$	6.57 × 10^−4^
$X_{a2}$	7.66 × 10^2^	$X_{b2}$	−9.04 × 10^−3^	$X_{c2}$	1.07 × 10^−2^
$X_{a3}$	4.65 × 10^2^	$X_{b3}$	7.94 × 10^−3^	$X_{c3}$	8.28 × 10^−3^
$X_{a4}$	6.86 × 10^3^	$X_{b4}$	1.49 × 10^−3^	$X_{c4}$	3.56 × 10^−2^
$X_{a5}$	2.76 × 10^2^	$X_{b5}$	−2.17 × 10^−2^	$X_{c5}$	5.73 × 10^−3^
$x_{a1}$	−2.34 × 10^3^	$x_{b1}$	−8.87 × 10^−4^	$x_{c1}$	4.81 × 10^−2^
$x_{a2}$	1.98 × 10^2^	$x_{b2}$	−3.78 × 10^0^	$x_{c2}$	7.44 × 10^0^
$x_{a3}$	1.58 × 10^3^	$x_{b3}$	−9.20 × 10^−2^	$x_{c3}$	9.13 × 10^−2^
$x_{a4}$	−7.51 × 10^3^	$x_{b4}$	−1.81 × 10^−2^	$x_{c4}$	9.42 × 10^−2^
$x_{a5}$	1.02 × 10^4^	$x_{b5}$	−6.85 × 10^−3^	$x_{c5}$	8.13 × 10^−2^
$x_{a6}$	5.61 × 10^2^	$x_{b6}$	1.25 × 10^−1^	$x_{c6}$	9.07 × 10^−2^
$Y_{a1}$	6.28 × 10^2^	$Y_{b1}$	7.48 × 10^−4^	$Y_{c1}$	1.14 × 10^−3^
$Y_{a2}$	1.06 × 10³	$Y_{b2}$	1.58 × 10^−3^	$Y_{c2}$	2.66 × 10^−3^
$Y_{a3}$	1.79 × 10^2^	$Y_{b3}$	3.82 × 10^−3^	$Y_{c3}$	5.66 × 10^−3^
$Y_{a4}$	3.33 × 10^3^	$Y_{b4}$	1.25 × 10^−2^	$Y_{c4}$	1.14 × 10^−2^
$y_{a1}$	1.51 × 10^16^	$y_{b1}$	7.02 × 10^−0^	$y_{c1}$	1.27 × 10^−1^
$y_{a2}$	1.27 × 10^3^	$y_{b2}$	−2.51 × 10^−2^	$y_{c2}$	4.29 × 10^−2^
$y_{a3}$	1.29 × 10^2^	$y_{b3}$	−7.33 × 10^−2^	$y_{c3}$	3.07 × 10^−2^
$y_{a4}$	4.34 × 10^2^	$y_{b4}$	−9.49 × 10^−2^	$y_{c4}$	4.96 × 10^−2^
$y_{a5}$	5.34 × 10^2^	$y_{b5}$	−1.79 × 10^−1^	$y_{c5}$	9.40 × 10^−2^

**Table 3:** Tsai–Wu model coefficients for 3D printed CF-PEEK.

Parameter	Values/MPa	Parameter	Values/MPa	Parameter	Values
$X_{T}$	103	$X_{C}$	110	$\mu_{xy}$	0.29
$Y_{T}$	54	$Y_{C}$	71	$\mu_{yz}$	0.40
$Z_{T}$	30	$Z_{C}$	146	$\mu_{xz}$	0.35
$S_{xy}$	40	$G_{xy}$	852		
$S_{xz}$	27	$G_{xz}$	532		
$S_{yz}$	30	$G_{yz}$	804		

## Data Availability

Data will be made available on request.

## Nomenclature

$X_{ai}$, $X_{bi}$, $X_{ci}$ $x_{ai}$, $x_{bi}$, $x_{ci}$	Linear fitting parameters of X-type specimen, index i varies from 1 to 6
$Y_{ai}$, $Y_{bi}$, $Y_{ci}$ $y_{ai}$, $y_{bi}$, $y_{ci}$	Linear fitting parameters of Y-type specimen, index i varies from 1 to 5
$X_{T}$, $Y_{T}$, $Z_{T}$	Tensile yield strength in the X, Y, and Z directions
$X_{C}$, $Y_{C}$, $Z_{C}$	Compressive yield strength in the X, Y, and Z directions
$S_{xy}$, $S_{yz}$, $S_{xz}$	Shear strengths in the XY, YZ, and XZ planes
$G_{xy}$, $G_{yz}$, $G_{xz}$	Shear modulus in the XY, YZ, and XZ planes
$\mu_{xy}$, $\mu_{yz}$, $\mu_{xz}$	Poisson’s ratio in the XY, YZ, and XZ planes
$F_{1}$, $F_{2}$, $F_{3}$	Linear stress term coefficients
$F_{11}$, $F_{22}$, $F_{33}$	Quadratic normal stress term coefficients
$F_{44}$, $F_{55}$, $F_{66}$	Quadratic shear stress term coefficients
$F_{12}$, $F_{23}$, $F_{13}$	Cross stress term coefficients
$E_{XT}$, $E_{YT}$,	Tensile modulus in X/Y-direction
$E_{XC}$, $E_{YC}$,	Compressive modulus in X/Y-direction
$E_{Z}$	Elastic modulus in Z-direction
$\rho_{e}$	Element design variable (density)
p	Stiffness interpolation penalty factor
q	Stress interpolation penalty factor
$E_{0}$	Stiffness of solid material
$\sigma_{TW,e}$	Tsai–Wu failure index for element
$\sigma_{PN}$	Global P-norm stress aggregation
C	Compliance (strain energy)
$B_{e}$	Strain–displacement matrix
$L_{e}$	Global-to-local displacement transformation matrix
$k_{e}$	Element stiffness matrix $k_{e}=E_{e}k_{0}$, where $k_{0}$ is solid element stiffness.
K	Global stiffness matrix
F	Global load vector
$D_{0}$	Material elasticity matrix
**λ**	Adjoint variable
$V_{f}$	Volume fraction constraint
P	P-norm aggregation parameter
